# Novel Psychoactive Substances in Custodial Settings: A Mixed Method Investigation on the Experiences of People in Prison and Professionals Working With Them

**DOI:** 10.3389/fpsyt.2020.00460

**Published:** 2020-05-26

**Authors:** Ornella Corazza, Sara Coloccini, Shanna Marrinan, Mike Vigar, Caryl Watkins, Cosimo Zene, Attilio Negri, Andreas Aresti, Sacha Darke, Raffaella Rinaldi, Antonio Metastasio, Giuseppe Bersani

**Affiliations:** ^1^Centre for Clinical & Health Research Services, School of Life and Medical Sciences, University of Hertfordshire, Hatfield, United Kingdom; ^2^Department of Medical-Surgical Sciences and Biotechnologies, Sapienza University of Rome, Rome, Italy; ^3^G4S Care and Justice Services, Swansea, United Kingdom; ^4^School of History, Religions and Philosophies, University of London (SOAS), London, United Kingdom; ^5^School of Pharmacology and Clinical Toxicology, University of Milan, Milan, Italy; ^6^History, Sociology and Criminology, University of Westminster, London, United Kingdom; ^7^Department of Anatomical, Histological, Forensic and Orthopedic Sciences Departmental Section of Legal Medicine, Sapienza University of Rome, Rome, Italy; ^8^Camden and Islington NHS Trust, London, United Kingdom

**Keywords:** novel psychoactive substances, spice drugs, prison system, violence, Synthetic Cannabinoid Receptor Agonists (SCRAs)

## Abstract

**Introduction:**

Novel Psychoactive Substances (NPS), especially Synthetic Cannabinoid Receptor Agonists (SCRAs), pose a substantial challenge to health and the security of the prison environment. This study analyses the phenomenon from the perspective of people in prison and that of professionals working with them.

**Methods:**

A phenomenological qualitative approach was used to analyze self-reported experiences with ‘Spice’ (NPS) among users in prison. A semi-structured questionnaire was also disseminated among professionals working in these settings to better understand (a) the impact of NPS on their work; (b) perceived issues on safety in their working environment; (c) approaches used to tackle the phenomenon and best practices.

**Results:**

Psychotic events resulting from the collected Spice accounts (5) were marked by hallucinations, depression, self-harm, and suicidal ideations. Other emerging elements included fear, paranoia, inability to be with others, mistrust, breakdown and other risky behaviors. Overall, 186 responses from prison staff were collected across the country. 67% claimed NPS to have had a deep impact on their work as they commonly witnessed espisodes involving outbursts of anger, slurred speech, hallucinations, psychosis, and significant mental deterioration among those in prison. Some 91% have witnessed aggression at least once, with 53% experiencing direct harm. Suggested interventions included enhanced training and education (84%), improved detection (92%) and treatment and support services (93%).

**Conclusions:**

Findings highlight the urgent need for joint multi-disciplinary efforts to tackle the exponential escalation of NPS in prisons as well as to facilitate the recovery and societal reintegration of those affected. Phenomenology can be recommended as a valuable methods to study drug induced experiences.

## Introduction

Novel Psychoactive Substances (NPS) pose ‘the most serious threat to the safety and security of the prison system’ (1). Emerging data from British prisons are of great concern with an estimate range between 33 and 90% of people in prison regularly using these substances, especially Synthetic Cannabinoid Receptor Agonists (SCRAs) (1, 2). NPS are typically analogs of other psychoactive substances, created in makeshift laboratories without any safety protocols or testing in humans, thus posing a severe health challenge for this vulnerable population and professionals working with them. Although prisons have comprehensive security systems in place to detect contraband items, NPS more easily evade standard detection methods, as they are often colorless, odorless and active in very small quantities. In addition to the usual methods of import such as hiding in bodily orifices, SCRAs have also been sprayed onto clothing, food items, letters and even children’s drawings sent into prisons. As the use of these drugs in prisons has reached alarming levels, so has the number of cases of self-harm, suicide attempts, aggressions and assaults, and ambulance call-outs (2–4). According to the most recent review of the Ministry of Justice, there were 308 deaths in custody in the year to September 2019. Incidents of self-harm have risen by 22% from the previous year and the number of assaults increased by 5% up to June 2019, with a rate of 451 episodes of violence per 1,000 prisoners (5). The use of NPS in prisons has also been associated with organized crime, bullying, debt and suicides (1, 6). Associated violence can often extend to the wider community; the family and friends of those who are experiencing addiction or accumulating debts in prison may also be pressured to provide funds or carry NPS into prison.

Overall, there are currently 118 prisons in England, each with a dedicated healthcare team for prisoners. In 2018–19, 53,193 adults received treatment in such secure settings (99% of which was in the adult prison estate) (7). Of these, 11% presented to treatment services for NPS related issues, either on their own or alongside other substances, up from 6% in 2015–16. Health risks associated with NPS use include convulsions, paralysis, tachycardia, psychosis, anxiety, depression, self-harm and suicide. This is due to their higher potency and affinity for the cannabinoid receptors CB1 and CB2 compared to THC. In particular, the regular use of SCRAs has been associated with aggressive behaviors and other psychotic symptoms, especially among those already affected by addiction or mental illness (8). More in detail, NPS use, and particularly SCRAs have been linked with the diagnoses of bipolar disorder, personality disorders and the onset of schizophrenia and related disorders (9). Acute and long term consequences of NPS consumption may have a number of important clinical implications, especially for young users (10, 11). Incidents involving NPS also carry a higher risk of hospitalization (88.9% of admission by drug with an emergency medical treating was using SCRAs) compared with traditional drugs (12). Furthermore, NPS use has been associated with a high prevalence of fatalities (13). These figures may also be under-estimated as NPS related fatal intoxications may be under-investigated due to difficult and expensive methods of post-mortem detection. Treatment is also challenging because of the potential interaction between NPS and prescription medications common in prison settings, such as benzodiazepines and other psychotropic medications (9). Because of the novelty of the phenomenon, training for health and other professionals working in such settings has not kept pace with evolving trends in drug use, resulting in a lack of confidence in some staff responding to NPS related emergencies (14, 15). This study aims evaluate the experiences of both people in prisons and professionals working with them in order to inform the development of new targeted responses. The study was approved by the University of Hertfordshire Ethics Committee (LMS/SF/UH/03868). Additional ethics approval was gained by the National Research Committee (NRC) for the secondary analysis of the accounts collected from Her Majesty’s Prison Service (HMP) & Young Offenders’ Institution *Parc*.

## Materials and Methods

Accounts from people in prison affected by NPS use were collected at Her Majesty’s Prison Service (HMP) & Young Offenders’ Institution *Parc*, which is Category B Training Prison in South Wales, operated by G4S Custodial and Detention Services. A selected number of individuals, known to the substance misuse team as users of SCRA, also known as ‘Spice’, were approached by the Drug Strategy Manager and informed about the study. Those who agreed to participate prepared a written account about their experiences of using ‘Spice’. This was written either on their own, or with the support of staff. The collected accounts have sometimes been edited for clarity, but strongly reflect the submissions of each of the individuals. These were analyzed according to a qualitative phenomenological approach focusing on the assessment of subjective experiences, through personal narratives, to trans-personal constructs (16). Phenomenology is an increasingly valued research methods in the context of clinical research for bringing forth the typical feature(s) of personal experiences. In our study, particular attention was given to the approach proposed by Paul Ricoeur, which emphasizes the conceptualization of the participant as an ‘agent’, a self among other selves, in an effort to experience freedom (17). Written informed consent was obtained for the publication of any potentially identifiable data included in this article.

In addition, a semi-structured questionnaire was designed and disseminated among professionals working in prisons and acute mental health settings *via* a previously established network of collaborators. The tool consisted of 19 questions on four different themes: (a) basic demographic information; (b) knowledge of NPS and impact on their work; (c) issues of perceived safety in the working environment; (d) approaches used to tackle the phenomenon and best practices. The questionnaire was created on Qualtrics, a cloud-based software developing online surveys that provides data analysis, sample selection, bias elimination, and data representation tools. Responses to the questionnaire were collected both in person and online and anonymized. Face-to-face interviews with a selected number of staff also took place. Research material was securely stored and shared only between members of the research team. A group of ex-offenders, currently undertaking a degree at the University of Westminster, was consulted at different stages in order to facilitate the development of the study material. All participants gave individual informed consent to participate in the study and further authorization was obtained from the heads of prisons where the questionnaire was disseminated.

## Data Analysis and Results Assessment

### Analysis of ‘Lived Experiences’ With NPS Among People in Prison

Five case studies were collected and analyzed according to a phenomological approach.

Case 1:

*“I first used Spice in 2013 in prison, my mate gave it to me. I didn’t know what it was but it blew my mind. The vents at the back of the cell were moving, it looked like a mouth so I thought they were talking to me. I moved to another prison and was just using it all the time. I got into debt, I was stressed out all the time and it didn’t help that I was in a cell with my Uncle who was pressuring me to get more and more. I ended up having a breakdown because of it all, and tried to throw myself off the 3s. Just before my breakdown I had a really bad trip. I saw a hole in the floor of my cell, and when I looked down into it I was pulled down and I am convinced I went to hell. There was a wrinkly dog that had a big studded collar on. I was shitting myself, really scared, and then my Uncle who has passed away since, pulled me back out and told me it wasn’t my time. Spice is great when you’re on it, when I’m off it’s the worst I’ve ever been. After I heard about my friend dying from it, it really shook me up. I realised I was 33 and had nothing to show for my life. I moved out of the cell with my Uncle and have been determined to stay away from it. I want more for myself.”* JP. 32 years old, White British, Level 1 Literacy, Entry 3 Numeracy

Analysis: JP shows a very high awareness of his experience with Spice, recalling vivid memories of the first time and most of all the ‘bad trip’, which seems to mark a turning point. He presents with a complex multimodality anomalies of perceptions where illusions and hallucinations (visual, audiotry and tactile) were mixing. The abnormalities of perception are mood congruent with his despair. The subject is dragged to a ‘dark hole’ that he identified with hell. Of particular relevance is also the second part of the experience, where the others in the story—the mate and the uncle intervened and saved him. This element is very important because although the contest of the perceptual abnormalities was the same, his experience changed with the introduction of the salvific role of the recently deceased uncle, who saved him from hell and therefore from the ‘bad trip’. When experiencing hell only others around us can offer a glimpse of hope in order to recover our true self. Despite all good intentions and determination, JP knows he needs a hand to overcome the cycle of stress, debt and breakdown which will inevitably take him back to hell. But his cry for help—wanting more for himself—cannot be silenced.

Case 2:

*“I’ve never felt so afraid ever in my life of dying. I think of loads of reasons why I shouldn’t take spice but it’s that massive cloud of rush that comes all over my whole body that keeps me saying ‘yes I want it’. It started 7 years ago. I’d been released from prison homeless and went into town to see who was about, and saw a group of boys making a spliff. I just thought it was skunk so didn’t ask any questions and just took 6 long drags and held it in. It was Christmas time so there were people everywhere and the paranoia kicked in. I was walking through crowds of people and everything was zooming in and out, the people and the buildings, and it was just like being in a weird bubble with everything echoing around me. That should have been the end. Spice is the only drug where I hear a voice saying ‘you’re going to die, you’re going to die’. That alone should be enough for me to stop smoking it but it’s not. But at the same time I don’t want to die. I certainly shouldn’t enjoy spice if that voice keeps telling me I’m going to die, and I honestly don’t want to leave prison in a body bag. But I can’t stop. Spice scares the shit out of me but when I get the urge to score some more, it’s not that I forget about the scary signs, I just make the effort to forget them. That scares me even more.”* GC. 35 years old, White British, Entry 1 literacy, entry 2 numeracy.

Analysis: The fear of death, in itself traumatic, is accentuated when experienced, as in GC’s case, under the influence of Spice. The awareness of finitude and fallibility is pushed by GC to its limit when recognising that he is able to push away the ‘scary signs’—the voice inside him—so as to satisfy ‘the urge’. This ability to forget, and in a way to trick himself, is indeed even more scary, as GC honestly admits: he is still struggling with this contradiction, despite the many reasons he can list to stop taking Spice. The phenomenological analysis also shows a significant change in the quality of the experience expressed by the evolution of the psychopathology. The individual’s symptoms starts, when using spice, with complex multimodal illusions (mainly visual) and evolve to audiotry hallucinations with a voice making terrifying remarks. Under the influence of the drugs, therefore, the patient has an experience similar to the one that individuals with schizophrenia have.

Case 3:

*“I started smoking Spice in 2016 in prison. Subby (Subutex, i.e. Buprenorphine) was my thing at the time, when I couldn’t get that I was told I was told smoking Spice would help with my withdrawals, so I started smoking small amounts of it but it wasn’t doing what my friend told me it would, so I started smoking much more. Bigger the pipe the better as it’s called in here. When Code Blueing (needing emergency health attention in prison), the only way I can describe it’s like going into an epileptic fit where you blank out and come around feeling really stoned, so being high like you would when you smoke weed, but if you’re not used to Spice you go into instant paranoia, you feel like all the walls are caving in on you, and when you lie on the bed it feels like you’re sinking into it. Smoking Spice took me to a really dark place, got to a point where I was so paranoid and thought everyone was out for me. I didn’t even feel safe walking to get my dinner and I lost 3 and a half stone in weight as I thought that people were putting poison in my food. The more I smoked the more in debt I got, and didn’t really care what would happen to me. It’s so addictive that getting more was all I cared about. I was even letting people give me happy slaps or punches to the face so I could get my fix. It was mentally draining, I was getting no sleep whatsoever, getting really bad cramps in my stomach. Seeing people smoke Spice goes through me as I know what it did to me. I fell out with my family as I was constantly asking for money, making up lies like saying my trainers had been robbed from the cell but really I’d sold them to get a bit of Spice. I got to the point where I’d sold everything in my cell, even my last shower gel and last toothpaste, I even sold my TV to someone on basic (basic regime on the prison IEP scheme), so that I could get high for the next hour and spent the rest of the day just looking at the walls. I’ve even drunk washing liquid from the washing machine to get Spice from someone. It ruined my life, I stopped speaking with my family for years because I chose to smoke Spice, it takes you to a really dark place where you have no respect for anyone and you don’t give a shit about yourself. At one point, I didn’t shower for three and a half months. When I last got out of jail I carried on smoking Spice and smoked a strong strand of Mamba with two friends, we all ended up in hospital, one of my mates died and the other ones got to wear a colostomy bag for the rest of his life. Luckily enough nothing bad happened to me but it made me take a long look in the mirror and I thought I need to change now or end up in a body bag and so since that happened I can’t even stand the smell of Spice. If anyone was thinking about starting to smoke Spice, I’d tell them to stay away from it, it ruined my life. I’m doing much better now, all my family are speaking to me, I’ve got my health back. So that’s my story of the Devil’s drug, Spice.”* RB. White British, 29 Years old, Entry Level 3 education literacy and numeracy.

Analysis: RB is the youngest of this group, but also the one who has gone all the way ‘to a really dark place’ (and ‘mentally draining’), and back: no respect for anyone or himself, falling out with his family and loved ones, on a clear path towards self-loathing and self-destruction and carrying on smoking Spice when out of jail. Only when a mate died and another one remained permanently injured he managed to ‘take a long look in the mirror’. Redemption is seen here as a process of self-reflexivity that awakens the inner-self to return to the original conditions of selfhood. The first sign of this is re-establishing ties with his family, being recognized and accepted again by them and, in the end, also being ‘forgiven’, as full part of the course of redemption.

Case 4:

*“I started smoking Spice in 2015 when I was in Cardiff Prison. I was introduced to it by my mate, I had no idea what it was before I took it. It numbed me and made me feel nice, but i know you can die from it which isn’t good. I was stressed and bored and I used to numb that, but I was getting stomach cramps because of it. I didn’t really have anybody around me looking out for me, my Dad died when I was young and I lost my girlfriend to an overdose, so there’s been a lot of trauma in my life and Spice blocks all that out. When I moved into a cell with somebody who was using all the time it dragged me down, but since moving in with my new padmate, I feel more encouraged to stay away from it, I’m back in the gym, I’ve got myself a job on the wing and I’m doing much better. My advice to anyone thinking of using Spice is don’t start it, because you can’t stop.”* LD. White British, 35 Years old, Entry Level 3 literacy and numeracy in education.

Analysis: LD’s story helps us to place his narrative in a wider scenario, beyond prison and prior to the encounter with drugs or Spice in particular. This is the case also in other accounts, but here the ‘trauma’ takes the shape of specific faces of loved ones—his father and his girlfriend—who are dearly missed. But blocking trauma with Spice is an illusion, and while some might influence negatively, others (even prison mates) encourage him to take a different path.

Case 5:

*“I used around 2012 or 2013, I was out in the car and gave a mate a lift. I was only taking him about half a mile. When my mate got in the car he was rolling up a spliff, he told me it was Spice and I’d heard a bit about it but never had it. I had a couple of puffs whilst we were driving and i got carried in the conversation and kept smoking without realising. Once he’d got out of the car I realised I couldn’t drive anymore, it was a really rapid onset, not as fast as when you’re pinnin’ but really quick. I dunno how much he’d put in there, but it affected me really badly. I started off feeling really relaxed, which is why I guess they link it to cannabis, but it intensifies really quickly. I had no control of my limbs, hands, arms, legs, couldn’t move them if i wanted to. luckily I was on my own so I didn’t feel too paranoid. I definitely would have panicked with people around me. After a while, I thought I was OK, so tried to drive, but quickly realised I was doing it really badly. I got stopped by the police a short while down the road, managed to convince them I was just really tired and after breathalysing me they let me go. Never used it since, and I’d never use it again. I’m a regular drug user, including heroin, but Spice really is the bottom of the barrel.”* MD. 56 years old. Level 2 literacy and numeracy. Mixed origin, British.

Analysis: One single experience by MD was enough to convince him to stay away from Spice. As an older and ‘regular drug user’ he was fully aware that Spice was ‘the bottom of the barrel’, given the paralysing effect of this drug. This account makes reference to the lesser detectability of SCRAs, which has contributed to their popularity.

### Survey Results Among Professionals Working in Prison

#### Study Sample

Overall 186 individuals (92 females; 93 males), aged 25–65, took part in the study. Participants belonged to different occupational sectors, including prison and probation services (70%), clinical and health care (15%), education (3%), social work/care (2%) and to a lesser extent to business administration, Government facilities, Public Health, Human Rights, and Police (**Table 1**). The majority of participants (76%) were working in a prison setting, 7% reported working in such environments predominantly, 8% sometimes, 8%’rarely or never worked in prisons; two participants had previously worked in prisons but no longer did so. 45% of the sample had worked in prisons for more than 10 years; 15% for 5–10 years, 15% for 2–5 years, 10% for 1–2 years; 7% of participants had 6–12 months experience. 8% had been working there for less than 6 months.

**Table 1:** Q. What is your occupational sector?

	Occupational sector	%
1	Clinical and Health care	15
2	Education	3.2
3	Social work and care	1.6
4	Prison and probation services	70.4%
5	Academia	0.5%
6	Other	9.1%

##### Impact of NPS on Staff Working Environment

The majority of respondents considered their work “largely affected” by NPS (81%). All participants agreed that NPS have a negative impact on their working environment. Operational instability, bullying, potential staff corruption, and time constraints were also mentioned as contributing factors during the interviews. Limited access to training opportunities was another influencing factor. A participant stated: “The amount of emergency health care codes makes the planned work too much difficult” (**Table 2**).

**Table 2:** Impact of NPS on staff working environment.

Issue	Description
Aggression/Violence	*« It is difficult to get people to listen, to respond. They may be agitated, unreasonable, aggressive. Their priorities are not around progress and growth »* *« Affects vary from adverse health effects, to violence related to debt (this includes being assaulted through not being able to pay off debt, being violent to others to pay off debt, or violent as a side effect to use). Self-harm also linked with inability to manage use of NPS. Use of NPS has impacted on men attending work or education, as well as gym sessions and programs. »* *« We have noticed Increases risk of self-harm and suicide attempts, increases risk of violence towards others. »* *« …Increased levels of violence, bullying others to use to test drug levels, medical issues, falling over, sliding down walls, no recollection of their behaviors. »*
Time constraints	***« The impact it has on users renders them unable to keep appointments and/or progress. It can also kill them. The spice use within the wings and the prison regime frequently leads to lock downs due to incidents, so again it’s impossible to keep appointments scheduled and many, many meetings have been cancelled. »*** *«It has impacted my ability to interview offenders for report and assessments. I have had to terminate these appointments and reschedule. NPS also affects the work that we are trying to do to rehabilitate offenders. It negates all the effects and support that is provided. »* *« We all noticed a failure to attend for interview for sentence planning. They are unable to work and get into debt, under threat … and many of them are coerced to do things. As a result, this is disruptive for the prison regime. »* *« It breaks up the normal day and the Prisoners who would have a full educational session only receive limited time with the Teacher/Instructor. »*
Safety	***« NPS lead individuals in a zombie like state requiring further staff intervention with regards to their safety. This in turn destabilizes the regime and causes problems for non-NPS inmates. NPS can also lead to aggressions towards staff and others and it is largely responsible for most of debts and bullying in prison. »*** *« It has made the pressure on support services increase. It has increased the level of debt. It has led to more severe and sometimes fatal health issues. It demotivates prisoners. It has made the prison environment less safe. »*
Health	*« There is a lack of concentration, a lack of motivation to do anything other than use NPS. »* ***«NPS have an impact on many things*. ** ***On their engagement*, ** ***on their physical health*, ** ***on their mental health*. ** ***A cycle of events that lead them to continue their use*. ** ***They can’t function*** ***They can’t work »*** *« Prisoners heavily using NPS can ‘fall apart’. They have no interest in personal hygiene, they often sell their food and of course lose weight rapidly»* *« Working with individuals with extreme NPS addictions is difficult even when they are not under the influence at the time, they are often forgetful and experience extreme mental health issues. NPS makes drug users experience a variety of health problems that also interfere with their engagement, to attend health care appointments or because they are generally unwell*. *I have encountered individuals who have had strokes due to NPS use and this has damaged their ability to engage with rehabilitation in prison due to a lack of specialized care for these types of long term conditions. »* *« Some prisoners have had to be transferred to psychiatric inpatient units. Instances whereby some have completed stopped eating due to paranoia that the food is poisoned, others have completely refused all medication, either to help manage their mental state as a result of NPS use or medication to manage their pre-existing health conditions, such as diabetics or epileptics. »*

#### Violence and Aggression

For the majority of the sample, outburst of anger, aggression and violence were of great concern. While 9% stated to have never seen any aggression, others witnessed it at least once or twice (9%), less than 10 times (18%), more than 10 times (28%) and more than 50 times (36%) (**Table 3**).

**Table 3:** Q. In the past year, have you seen any episodes of violence or aggression within the prison setting?

#	Answer	%
1	More than 50 times	36%
2	More than 10 times	28%
3	Less than 10 times	18%
4	Once or twice	9%
5	No never	9%

Overwhelmingly, respondents thought that NPS were the main cause for such episodes. While 47% have never experienced direct harm, such as an assault, 13% declare to have been a victim of it (or to know a colleague with such a history behind) on one occasion, 21% between two and five times, 19% more than five times (**Table 4**).

**Table 4:** Q. Have you or a colleague (in your presence) ever experienced direct harm, such as an assault, which you believe to be related to the use of NPS?

#	Answer	%
1	Yes on multiple occasions (more than five times)	19%
2	Yes, between two and five times	21%
3	Yes on one occasion	13%
4	No, never	47%

#### Substances

**Figure 1:**
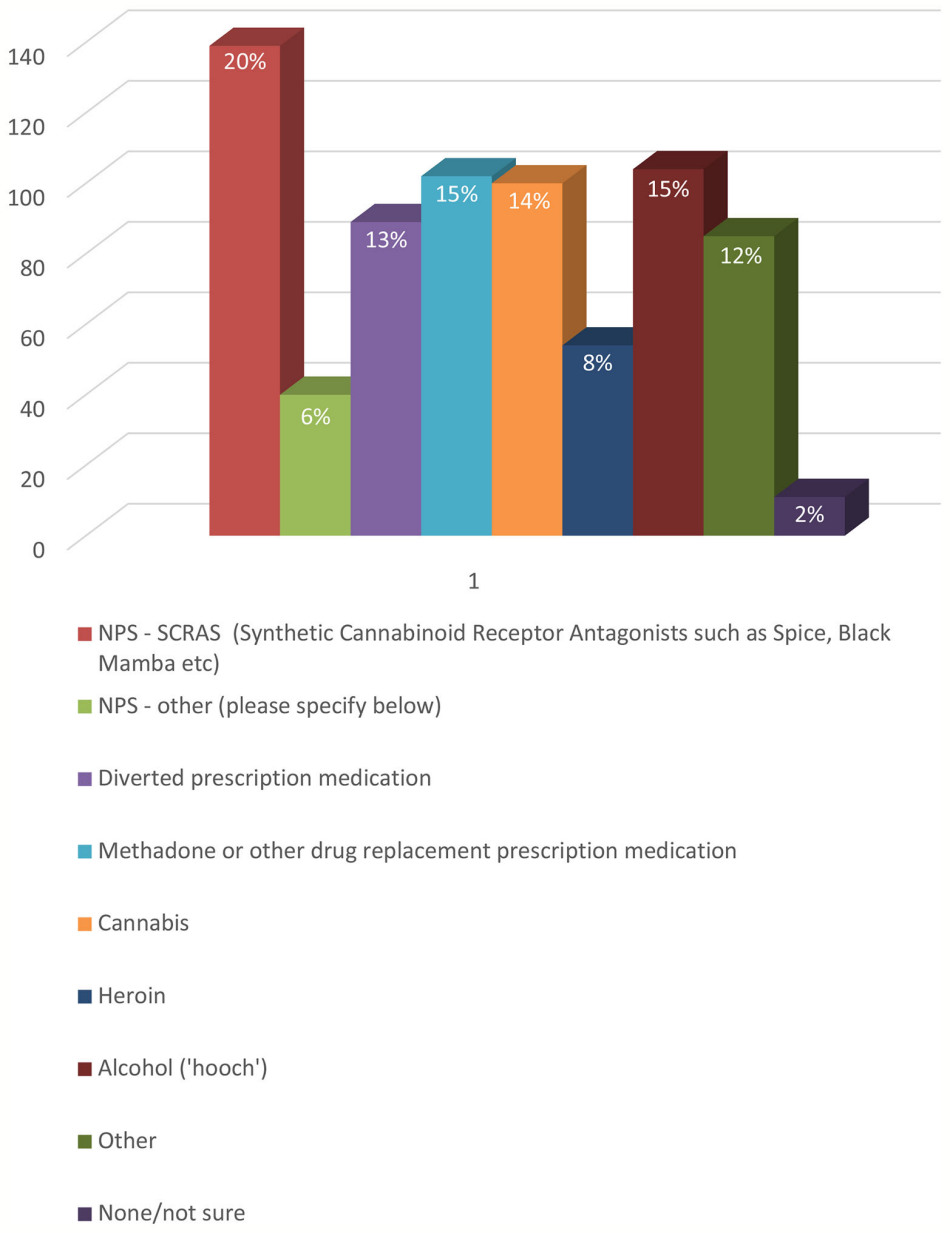
Q. Are you aware of one or more prisoners using one of the following substances?

Survey participants reported witnessing, or being otherwise aware of, use of a number of other substances in the prisons in which they work (**Figure 1**). These included cannabis (14%), heroin (7%), alcohol (14%) and also drug replacement medication or other prescription drugs, such as methadone, gabapentin, pregabalin, «Oids» (steroids), buprenorphine, and painkillers (14%).

During the interviews, staff also mentioned the deliberate consumption of products with the intention of inducing an intoxicated state. These included rat poison, alloy wheel cleaner, and bug killer repellant among others. It is not clear whether these are examples of ‘urban myths’ or hear-say, or first-hand observations. The likelihood of a person in prison being able to access some of these items is remote. “I work on a substance misuse induction wing and a recovery wing as an SMS worker so see and deal with a lot of hardened substance users. There is a regular supply of drugs with “Throw overs” and new receptions although there is now a body scanner in place so the new reception supply has reduced. The prisoners will use any form of NPS largely without questioning. Valium is very popular at the moment and buprenorphine snorting and concealment is popular although the prison is very restrictive with its issue. There is a lot of pressure on prisoners who are in receipt of ADHD meds to conceal them and pass them on. Tablets like Pregablin are regularly chased by prisoners seeking additional drugs or to conceal and sell on”.

**Figure 2:**
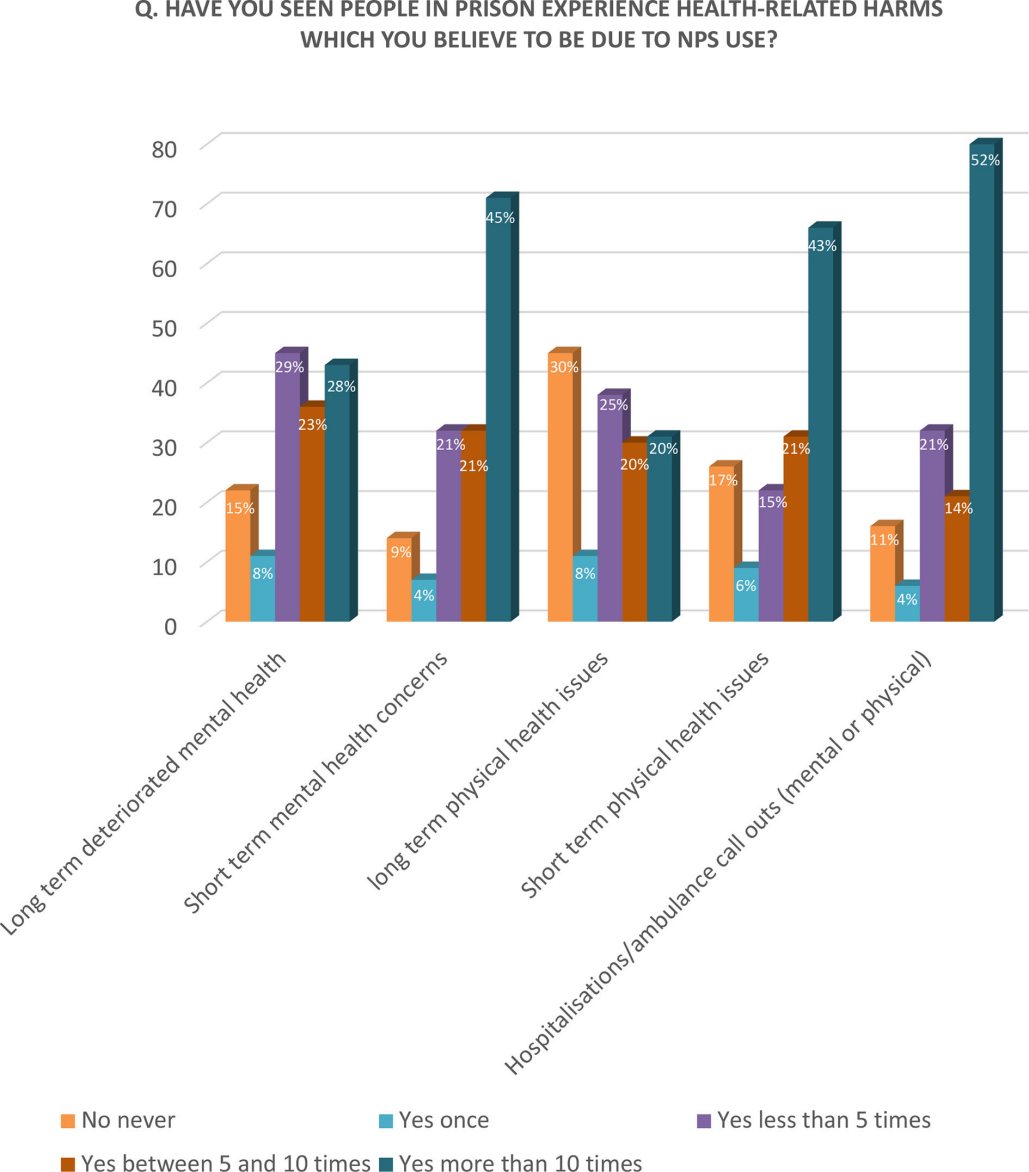
Q. Have you seen people in prison experience health-related harms which you believe to be due to NPS use?

Consumption of these drugs was related by the participants to a considerable increase in the number of ambulance callouts and hospitalizations that they witnessed (more than 10 times for 52% of the sample; see **Figure 2**).

#### Safety

Although the large majority of the sample felt ‘reasonably’ (51%) or ‘completely safe’ (13%), 25% felt ‘unsafe at times’ and 5% ‘often unsafe’. Some participants (6%) were not aware of the level of risks they were facing (**Table 5**).

**Table 5:** Q. Do you feel personally safe in your work in prisons?

#	Answer	%
1	Yes, completely safe	13%
2	Yes, reasonably safe	51%
3	I don’t know	6%
4	Unsafe at times	25%
5	Unsafe often	5%

All the participants agreed that NPS has a negative impact on their safety with 37% of them considering the risk ‘extremely high’, 38% ‘high’ and 19% ‘substantial’. Only 6% found a marginal impact on their safety (**Table 6**).

**Table 6:** Q. To what extent do you feel the use of NPS affects the safety of those working in prisons?

#	Answer	%
1	No affect	0%
2	A small effect	6%
3	A substantial effect	19%
4	A large effect	38%
5	An extremely large effect	37%

##### Health-Related Harms

As shown in **Figure 2**, the most observed health-related harms were ‘short-term mental health issues’, such as hallucinations, psychosis, slurred speech, and a significant mental deterioration. 45% of the sample dealt with such issues more than 10 times in their life, 21% between five and 10 times, 21% less than 10 times and 4% only once; while 9% none. Half of the interviewees reported to have witnessed more than 10 hospitalizations or ambulance call-outs due to NPS. A clinician observed: “NPS use contributes to the worsening of existing psychosis and to the presentation of new psychotic episodes”.

Less common were ‘short-term effects on physical health’, such as seizures or tachycardia, and long-term mental health issues. It is interesting to notice that 29% of interviewees have ‘never observed long-term physical health problems’ among inmates using NPS.

The main acute clinical consequences which were related to the consumption of NPS were represented by both ‘severe mental disturbances’, such as consciousness alterations or psychotic symptoms onset or exacerbations, and ‘disrupting behavioral abnormalities’, such as mostly dyscontrol, aggression and violence. These symptoms are not unique to any diagnosis, and often residents displaying these behaviors do not neatly satisfy the diagnostic criteria for any condition (such symptoms may be common in several mental disorders, or acute states). Such evidence confirms that a wide range of potential aspecific and mixed psychotic, affective and behavioral responses are reported as a result of NPS consumption. A general lack of self-care and personal hygiene was also perceived, as a healthcare professional stated: “*Prisoners heavily using NPS can ‘fall apart’. They have no interest in personal hygiene, they often sell their food and of course lose weight rapidly”*.

As a participant stated: “Patients are referred into mental health clinical settings following the use of NPS in prison. Diagnosis and management of presentation is increasingly difficult along with associated risks of violence and aggression for prolonged periods. Current clinical solutions are ineffective for longer periods until the substances begin to clear from the patient which can take several weeks. Until this time, medications have little clinical effect and there are no adequate means of containing disturbed behavior, even within Psychiatric Intensive Care Unit”.

Concerns for their own health also emerged, including risks related to the secondary exposure to potentially highly toxic substances, as NPS are introduced to prisons *via* drones, letters and other hidden channels, such as food deliveries and children’s drawings.

#### Approaches Used to Tackle the Phenomenon and Best Practices

A number of suggested approaches for managing NPS in prisons were rated by participants using a 5-point Likert scale.

##### Knowledge and Education Needs

Some 59% of the respondents rated their knowledge on NPS ‘sufficient’ (**Table 7**), but they strongly highlighted the need for training and more information on NPS, mainly to be delivered *via* face-to-face workshops (29%), conferences (19%), newsletters (17%), scientific articles (10%), online programs (9%), among others (**Table 8**). Only 3% indicated the use of mobile phone app as a preference, possibly due to the limited use of the tool in such high-security settings.

**Table 7:** Q. How would you rate your knowledge and awareness of NPS?

#	Answer	%
1	Excellent	23%
2	Sufficient	59%
3	Not sure	7%
4	Somewhat insufficient	9%
5	Minimal	2%

**Table 8:** Q. If you were to learn more about NPS and the implications for your work, how would you prefer to receive this information?

#	Answer	%
1	Conference attendance	19%
2	Webinar	3%
3	Face-to-face workshops	29%
4	Online CPD program	9%
5	Email bulletin/Newsletter	17%
6	Reference Website	7%
7	Mobile App	3%
8	SMS with updates/trends	3%
9	Scientific Literature/Academic articles	10%

##### Improving Current Detection Tools

**Figure 3:**
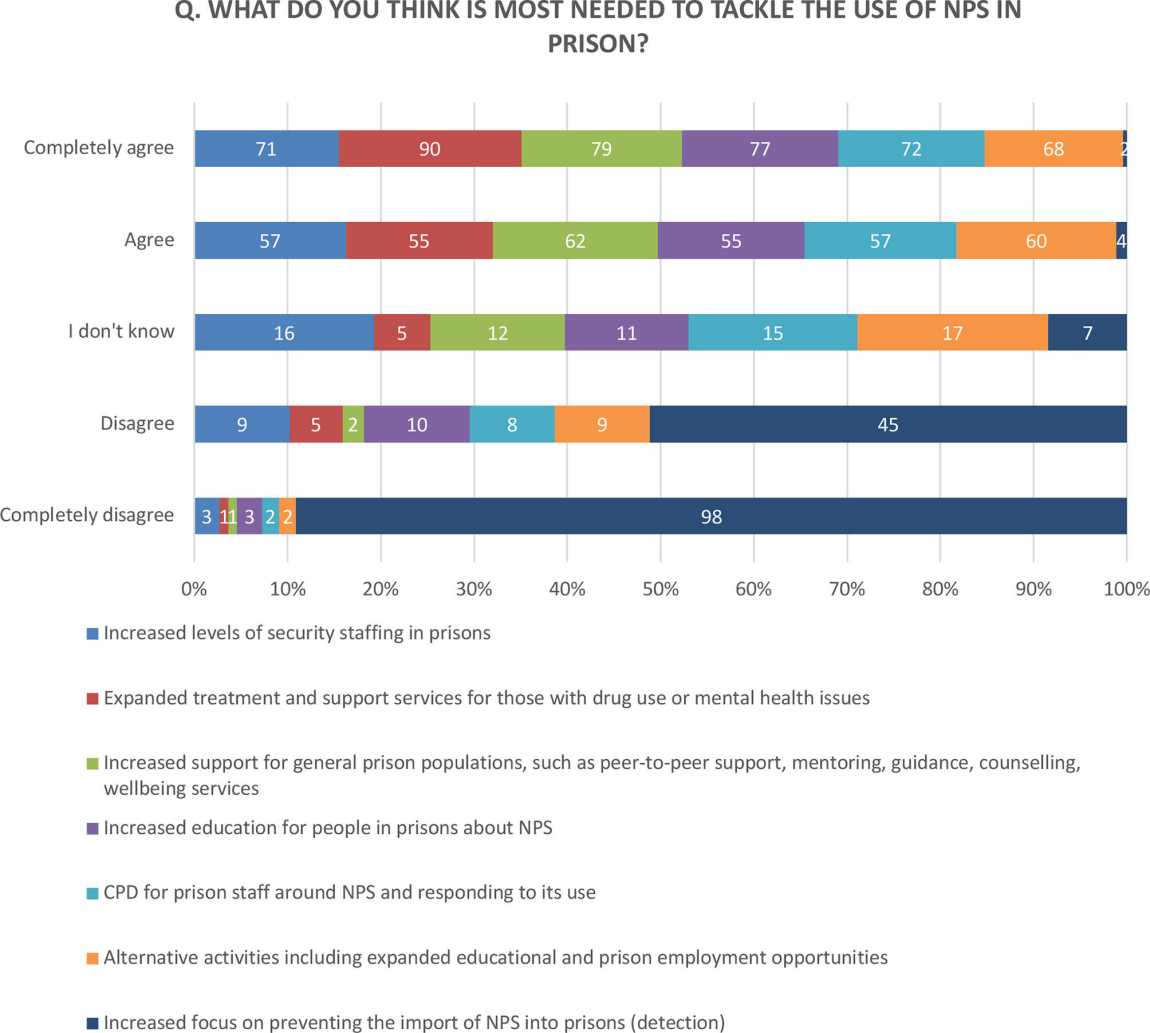
Q. What do you think is most needed to tackle the use of NPS in prison?

The majority of interviewees (92%) reported a need for ‘improving current detection tools’ (see **Figure 3**).

##### Expanding Treatment and Support Services for Those With Drug Addiction or Mental Health Problems

Some 93% expressed the need for ‘expanding treatment and support services for those with drug addiction or mental health problems’. They found that a better understanding of the underlying psychopathological issues will help the development of better assessment tools and both pharmacological and non-pharmacological treatment of those affected.

Another major barrier to treatment was the fact that inmates using NPS often refused to participate in rehabilitation activities, making their attempts even harder. It was claimed that negative and disruptive behaviors often sabotaged the therapeutical bond between prisoners and staff. A respondent said: “I work in a medium secure unit. Many of the patients use NPS. This makes it difficult for them to sustain relationships with staff, or engage full in psychological therapy. It affects patients’ mental states negatively, and slows up discharge”.

##### Offering Alternative Activities Including Expanded Educational and Prison Employment Opportunities

Making available ‘better education programs for people in prisons about NPS’ was found essential by the large majority (84%) of the participants. Some found that the prison environment often restricted offenders’ access to learning opportunities. According to them, training sessions were often cancelled due to ‘Spice’ incidents and staff being assaulted.

The development of Continuing Professional Development (CPD) courses for prison staff around NPS was also strongly supported (84%). As a participant stated: “The answer lies within prevention—we need to do all we can to prevent these items coming in. If we can achieve that or significantly reduce that—we have a better chance of getting these men clean and substance free and then we can work on interventions—i.e. substance misuse awareness, better education”.

##### Increasing Support for General Prison Populations, Such as Peer-To-Peer Support, Mentoring, Guidance, Counselling, Wellbeing Services

About 82% also encouraged the provision of additional learning and employment opportunities for people in prisons.

## Discussion and Conclusion

In a situation of emergency, our work provides a ‘first-hand’ perspective on ‘Spice’ (NPS) as experienced by people in prison in combination with the views, the concerns and the recommendations for best practices from professionals working with them. The phenomenological analysis of the five accounts shared by Spice users in prison allowed us to gain original insights on the underlying ‘feeling of paranoia’, such as the inability to be with others and the mistrust towards them. In one case the paranoia was so deep to generate prolonged periods of discomfort resulting in a complete breakdown. Psychological (and possibly physical) dependence was another common feature that emerged from our analysis. This was associated with unpleasant withdrawal symptoms. Psychotic outbreaks were marked by hallucinations, depression, suicidal ideations. Other negative experiences included self-harm, fear of death or seeing others dying or losing a friend. Even the language used to describe the experience was charged with negative connotations, such as: ‘going to hell’ (a hole in the floor), a ‘dark place’, hearing voices predicting one’s death, ‘the Devil’s drug’, and ‘bottom of the barrel’. Taking Spice with others was perceived also as a way to overcome boredom due to lack of engaging and stimulating activities. Interestingly, some therapeutical elements could also be found in the narrative of these experiences. For instance, in the first case (JP), the hallucinatory experience changed from ‘despair of hell’ to ‘redemption’ mediated by a savior represented by the recently deceased uncle. This allows us to confirm the validity of the phenomenological approach to uncover the more hidden aspects of a person’s inner world that would not be accessible otherwise and that could be used in future applications to address trauma and other causalities that perpetuate the misuse allowing new cues to emerge. Further, all the collected ‘lived experiences’ were part of a wider narrative, or “life-story”. The residents’ willingness to share their stories with Spice reflects a positive attitude aimed to regain the (self-)esteem and the recognition granted to them by the external community, which should be encouraged during the rehabilitation process. These findings well integrate with the results that emerged from the survey and the interviews with a wide range of professionals working in custodial settings. These helped us to shed new insights on the impact of NPS on their working environments, compared to that previously posed by other non-NPS substances. Although the majority of the sample had not been personally, or directly, affected by episodes of violence, a number of concerns were reported. Participants referred to their struggle to deal with the violent behavior of residents, resulting in aggressions between them, or towards staff members. Despite various mechanisms being in place, they highlighted the need for specialist training for those expected to respond to medical or welfare emergencies arising from use of NPS. Their unwanted exposure to potentially lethal or highly toxic substances was also emphasized. During the interviews, it was noticed that NPS were often misleadingly labeled by them as ‘Spice’, when in fact the term refers to only one category of NPS, SCRA and does not include a wide range of other substances. This discordance in understanding of what we mean by NPS constitutes a further challenge for clinicians and paramedics, since, as they stated, “they might be unaware of what they have taken and how”. As a result, a “treat what you see” approach has been adopted, and while still being the most indicated approach in acute medicine, the full implications of this, along with all potential drug interactions with other medications, are not fully understood. The challenge of responding to drug-related psychotic episodes, and the variability and the unpredictability of acute symptoms were other issues of concern that emerged from our study. These did not differ substantially from those commonly observed outside of custodial settings, such as emergency departments, where reported cases are characterized by clinical signs of acute mental intoxication with a mixed symptom profile, not meeting the psychopathological criteria of any specific mental disorder. However, this plays an even more serious role in the prison environment, increasing the perception of risk and difficulty to manage situations by the staff. As previous argued (8), the accurate collection of data on the use of NPS, investigations, treatment, violence pre-admission, violence during admission, length of stay, and readmission should strongly be reinforced at this challenging time. Finally, most of the interviewees strongly emphasized the need for more educational programs to raise risk awareness on NPS and facilitate their responsiveness to emergency situations. This may play a major role to reduce threats to the health of consumers, particularly with the appearance on the illicit market of new highly potent compounds which may escape traditional screening tests, such as Novel Synthetic Opioids (NSOs) (18). In conclusion, the unprecedented outbreak of NPS in British prisons exerted a negative influence on the overall psychological atmosphere in such environments, enhancing the general level of alarm and unsafety. Joint multi-disciplinary efforts are required to tackle the phenomenon and facilitate recovery and reintegration into society.

The study presents various limitations. As far as we know, this is the first time that a phenomenological approach has been used to analyze drug related experiences. A small sample (5) was selected for the investigation, which considering the positive results could be further expanded in the future. Strict inclusion or exclusion criteria were not provided for the selection of both our samples. Furthermore, we did not investigate the type of prisons nor the sociodemographic of the cohort. This may have led to potential bias in the selection of the respondents. In terms of future research, a larger study cohort would allow for the consideration of differences between the occupational sectors involved in terms of level of awareness, personal experiences, feelings and needs perceived, and of what has changed in professional practice. It is hoped that this study could help to build a clearer picture of where already-stretched resources may need to be focused and further extended to capture data from a greater number of prisons, from a more diverse professional cohort, and from wider territories, in particular beyond the United Kingdom, where the phenomenon is probably under-reported. The involvement of people in prison is also strongly encouraged in future studies as well as the consultation with a group of ex-prison residents to facilitate their implementation. Only a major joint effort among different key stakeholders can tackle the emergency caused by the spread of NPS in prisons and lead to the development of effective treatment and rehabilitation measures.

*“I am a real person with a real experience. Studies can be done until we are blue in the face. Let’s act now”*. A prisoner from HM Prison Guys Marsh (Dorset, England), August 2018.

## Data Availability Statement

The raw data supporting the conclusions of this article will be made available by the authors, without undue reservation.

## Ethics Statement

The studies involving human participants were reviewed and approved by University of Hertfordshire. The patients/participants provided their written informed consent to participate in this study.

## Author Contributions

OC designed and coordinated the study and the preparation of this manuscript. SC contributed to the data collection and analysis. SM and AN contributed to the literature review and the data collection. MV and CW collected the five case studies from prisons. CZ and AM carried out the phenomenological analysis. AA and SD coordinated the advisory group of ex-offenders. RR contributed to the literature review and provided clinical input. AA contributed to the phenomenological analysis and provided clinical input. GB contributed to the coordination of the overall project.

## Conflict of Interest

The authors declare that the research was conducted in the absence of any commercial or financial relationships that could be construed as a potential conflict of interest.

The reviewer GM declared a past co-authorship with one of the authors, OC, to the handling editor.

## References

[1] HM Inspectorate of Prisons (2015). HM Chief Inspector of Prisons for England and Wales Annual Report 2014-15. HM Inspectorate of Prison

[2] Ministry of Justice Safety in Custody Statistics, England and Wales (2019). Deaths in Prison Custody to September 2019 Assaults and Self-harm to June 2019. 31 October 2019.

[3] Ralphs R Williams L Askew R Norton A Adding spice to the porridge: The development of a synthetic cannabinoid market in an English prison. Int J Drug Policy (2017) 40:57–69. 10.1016/j.drugpo.2016.10.003 27955961

[4] Corazza O Roman-Urrestarazu A Handbook of Novel Psychoactive Substances. What Clinicians Should Know about NPS. 1st Edition London: Routledge (2018).

[5] Abdulrahim D Bowden-Jones O on behalf of the NEPTUNE Expert Group Guidance on the Management of Acute and Chronic Harms of Club Drugs and Novel Psychoactive Substances Vol. 2015 . London: Novel Psychoactive Treatment UK Network (NEPTUNE) (2015).

[6] The Prisons and Probation Ombudsman (2015). New psychoactive substances: Learning Lessons Bulletin - Fatal incident investigations. 1–5.

[7] Public Health England (PHE) (2020). Alcohol and Drug treatment in secure settings 2018-2019: report. Available at https://www.gov.uk/government/publications/substance-misuse-treatment-in-secure-settings-2018-to-2019/alcohol-and-drug-treatment-in-secure-settings-2018-to-2019-report.

[8] Shafi A Gallagher P Stewart N Martinotti G Corazza O The risk of violence associated with novel psychoactive substance misuse in patients presenting to acute mental health services. Hum Psychopharmacol: Clin Exp (2017) 32(3). 10.1002/hup.2606 28631373

[9] Acciavatti T Lupi M Santacroce R Aguglia A Attademo L Bandini L Novel psychoactive substance consumption is more represented in bipolar disorder than in psychotic disorders: A multicenter-observational study. Hum Psychopharmacol (2017) 32(3):1–6. 10.1002/hup.2578 28517032

[10] Martinotti G Lupi M Acciavatti T Cinosi E Santacroce R Signorelli MS Novel psychoactive substances in young adults with and without psychiatric comorbidities. BioMed Res Int (2014) 30(4):295–301. 10.1155/2014/815424 PMC4123484 25133182

[11] Martinotti G Cinosi E Santacroce R Papanti D Pasquini A Mancini V Substance-related psychopathology and aggressiveness in a nightlife holiday resort: Results from a pilot study in a psychiatric inpatient unit in Ibiza. Hum Psychopharmacol (2017) 32(3):e2586. 10.1002/hup.2586 28557062

[12] Winstock AR Global Drug Survey (GDS) 2019 https://www.globaldrugsurvey.com/wp-content/themes/globaldrugsurvey/results/GDS2019-Exec-Summary.pdf.

[13] Corkery JM Schifano F Martinotti G How deaths can help clinicians and policy-makers understand the risks of nove psychoactive substances. Br J Clin Pharmacol Special Issue: New Psychoactive Substances (2020) 86(3):482–98. 10.1111/bcp.14183 PMC7080619 31770457

[14] Public Health England (PHE) (2017). New Psychoactive Substances (NPS) in prisons: A toolkit for prison staff. Public Health England

[15] Criminal Justice Joint Inspection (2017). New Psychoactive Substances: the response by probation and substance misuse services in the community in England Crown copywright.

[16] Stanghellini G Ballerini M Qualitative analysis. Its use in psychopathological research. Acta Psychiatr Scand (2008) 117(3):161–3. 10.1111/j.1600-0447.2007.01139.x 18271797

[17] Ricoeur P (1960). Fallible Man, The Symbolism of Evil.

[18] Marchei E Pacifici R Mannocchi G Marinelli E Busardò FP Pichini S New synthetic opioids in biological and non-biological matrices: A review of current analytical methods. TrAC Trends Analyt Chem (2018) 102:1–15. 10.1016/j.trac.2018.01.007

